# Application of single-level and multi-level modeling approach to examine geographic and socioeconomic variation in underweight, overweight and obesity in Nepal: findings from NDHS 2016

**DOI:** 10.1038/s41598-019-56318-w

**Published:** 2020-02-12

**Authors:** Nipun Shrestha, Shiva Raj Mishra, Saruna Ghimire, Bishal Gyawali, Pranil Man Singh Pradhan, Dan Schwarz

**Affiliations:** 1 Institute for Health and Sport (IHeS), Victoria University, Melbourne, Australia; 2 Nepal Development Society, Chitwan, Nepal; 3 Department of Sociology and Gerontology and Scripps Gerontology Center, Miami University, Oxford, OH USA; 4 Section of Global Health, Department of Public Health, University of Copenhagen, Copenhagen, Denmark; 5 Department of Community Medicine and Public Health, Institute of Medicine, Tribhuvan University, Kathmandu, Nepal; 6 Nyaya Health Nepal, Kathmandu, Nepal; 7 Ariadne Labs, Harvard T.H. Chan School of Public Health and Brigham and Women’s Hospital, Boston, MA USA; 8 Division of Global Health Equity, Department of Medicine, Brigham and Women’s Hospital, Boston, MA USA; 9 Department of Medicine, Harvard Medical School, Boston, MA USA

**Keywords:** Health policy, Epidemiology

## Abstract

Nepal’s dual burden of undernutrition and over nutrition warrants further exploration of the population level differences in nutritional status. The study aimed to explore, for the first time in Nepal, potential geographic and socioeconomic variation in underweight and overweight and/or obesity prevalence in the country, adjusted for cluster and sample weight. Data came from 14,937 participants, including 6,172 men and 8,765 women, 15 years or older who participated in the 2016 Nepal Demography and Health Survey (NDHS). Single-level and multilevel multi-nominal logistic regression models and Lorenz curves were used to explore the inequalities in weight status. Urban residents had higher odds of being overweight and/or obese (OR: 1.89, 95% CI: 1.62–2.20) and lower odds of being underweight (OR: 0.81, 95% CI: 0.70–0.93) than rural residents. Participants from Provinces 2, and 7 were less likely to be overweight/obese and more likely to be underweight (referent: province-1). Participants from higher wealth quintile households were associated with higher odds of being overweight and/or obese (P-trend < 0.001) and lower odds of being underweight (P-trend < 0.001). Urban females at the highest wealth quintile were more vulnerable to overweight and/or obesity as 49% of them were overweight and/or obese and nearly 39% at the lowest wealth quintile were underweight.

## Introduction

The prevalence of overweight and obesity has increased dramatically during the past four decades while underweight rates has decreased, posing a major public health challenge both in developing and developed countries. In 2016, the World Health Organization (WHO) estimated more than 1.9 billion adults aged 18 years and older were overweight, and of these over 650 million adults were obese. Globally, the estimated prevalence of overweight increased from 21.5% in 1975 to 39% in 2014, and the prevalence of obesity nearly tripled from 4.7% in 1975 to 13.1% in 2014^1^. Over the same time span, the prevalence of underweight adults fell from about 14% to 9%^2,3^.

The dual burden of underweight and overweight and/or obesity is an emerging challenge for many low- and middle-income countries (LMICs), including Nepal^3–5^. The United Nations Food and Agriculture Organization estimates that globally 815 million people mostly in low income countries suffer from chronic undernutrition ^6^. Nepal has a chronic problem of undernutrition, but lately, overweight and/or obesity has emerged as an important public health concern^7^. Nepal’s population, estimated at 26.5 million as of 2011, is projected to rise to 30.4 million by 2021^8^. This escalating population growth coupled with less impressive economic growth in Nepal raises a grave concern in the context of ensuring safe and sufficient food supply to the growing population and thus the problem of chronic undernutrition may be further aggravated. According to STEPS Survey Nepal 2013, 8.8% of the Nepalese adults aged 15–69 years were underweight^9^. It was estimated that around half (54%) of the Nepalese population were suffering from chronic food insecurity and the situation is particularly worse in western mountainous regions^10^.

Sedentary life style and increased access to processed food, especially in urban areas, has resulted in substantial growth in overweight and/or obesity^4,5,11^. A survey in 2013 estimated that nearly 21% of Nepalese adults aged 15–69 years were overweight and/or obese. The same survey showed geographical discrepancies in prevalence of overweight and/or obesity with higher prevalence among residents of urban areas and hills^12^.

Previous studies using Nepal Demography and Health Survey (NDHS) 2016^13,14^ showed variation in underweight and overweight and/or obesity by individual level factors, i.e., women compared to men, urban residents compared to rural residents, and wealthy individuals were more likely to be overweight and/or obese. Although individual characteristics play an important role in manifesting health outcomes, including weight status, recent evidence suggest that health is also determined by population level characteristics such as residence, neighborhood’s walkability, availability of food and so on. Previous studies have extensively focused on individual characteristics associated with weight status among Nepalese population^13,14^ and less is known about the geographic variations in overweight/obesity burden as well as how much of the variation is explained by individual and geographic factors. Furthermore, socioeconomic factors are known to contribute to the geographic variation in other health outcomes^15,16^ and plausibly may contribute to the geographic variation in underweight and overweight and/or obesity. Yet, there is a paucity of data currently describing these upstream etiologies. If such an association is found, such data could provide insights to policy makers and program implementers to better understand the relationships between socio-economic, geographic, and nutritional variances.

This study aims to address this data gap by analyzing variation in underweight and overweight and/or obesity by indicators of geographic residence and socioeconomic indicators, using the nationally representative data from the NDHS 2016.

## Results

### Prevalence of underweight and overweight and/or obesity

A total of 14,937 participants (41.3% of men and 58.7% of women) from Nepal’s 77 districts, across seven provinces, were included in the analyses. The prevalence of underweight and overweight and/or obesity was 19.2% and 18.2%, respectively. Based on the Asian cutoff for Body Mass Index (BMI >22.9 kg/m^2^), the prevalence of overweight and/or obesity was 31.4%. In bivariate analyses (Table 1), participants age, sex, educational level, marital status, and household wealth quintiles and geographic location of their residency were associated with their weight status. A higher proportion of the youngest (15–25 years) and the oldest (55 years and above) participants wereunderweight whereas participants of middle age (25–54 years) were overweight. A higher proportion of females, those with no education or only preschool education, and living in urban areas were underweight (Table 1).

**Table 1:** Socio-demographic characteristics of study participants based on BMI status (underweight, normal weight, overweight and/or obesity) (N = 14,937).

	Underweight (<18.5 kg/m^2^) N = 2,864, 19.2%		Normal weight (18.5–24.9 kg/m^2^) N = 9,358, 62.7%		Overweight and/or obesity (>=25 kg/m^2^) N = 2,715, 18.2%		p-Value
	n	% (95%CI)	n	% (95%CI)	n	% (95%CI)	
**Age**							
15–25	1079 (37.9)	37.9 (36.0–39.9)	2893 (30.7)	30.7 (29.5–31.9)	277 (10.2)	10.2 (8.7–11.6)	<0.0001
25–35	355 (12.7)	12.7 (11.2–14.3)	1970 (21.2)	21.2 (20.2–22.3)	727 (27.6)	27.6 (25.6–29.6)	
35–45	289 (9.7)	9.7 (8.4–11.0)	1499 (15.9)	15.9 (15.1–16.8)	725 (27.2)	27.2 (24.9–29.4)	
45–55	284 (10.1)	10.1 (8.9–11.3)	1245 (13.0)	13.0 (12.2–13.8)	511 (18.1)	18.1 (16.4–19.8)	
55–65	368 (12.1)	12.1 (10.7–13.6)	957 (10.2)	10.2 (9.5–11.0)	306 (10.8)	10.8 (9.4–12.3)	
>65	489 (17.4)	17.4 (15.6–19.1)	794 (8.9)	8.9 (8.1–9.6)	169 (6.2)	6.2 (5.1–7.2)	
**Sex**							
Male	1166 (40.5)	40.5 (38.6–42.4)	4013 (44.0)	44.0 (42.8–45.1)	993 (36.6)	36.6 (34.9–38.3)	<0.0001
Female	1698 (59.5)	59.5 (57.6–61.4)	5345 (56.0)	56.0 (54.9–57.2)	1722 (63.4)	63.4 (61.7–65.1)	
**Education**							
No education, preschool	1339 (47.5)	47.5 (45.1–50.0)	3464 (36.8)	36.8 (35.1–38.5)	838 (30.2)	30.2 (27.8–32.6)	<0.0001
Primary	413 (15.1)	15.1 (13.7–16.5)	1536 (16.4)	16.4 (15.3–17.4)	487 (17.7)	17.7 (15.9–19.5)	
Secondary	887 (29.7)	29.7 (27.5–31.8)	3089 (32.7)	32.7 (31.3–34.1)	912 (33.5)	33.5 (31.2–35.9)	
Higher	225 (7.7)	7.7 (6.3–9.1)	1269 (14.1)	14.1 (12.8–15.5)	478 (18.5)	18.5 (16.4–20.6)	
**Residency**							
Urban	1666 (53.9)	53.9 (47.7–60.1)	5760 (59.2)	59.2 (54.5–63.8)	2013 (74.2)	74.2 (69.9–78.6)	<0.0001
Rural	1198 (46.1)	46.1 (39.9–52.3)	3598 (40.8)	40.8 (36.2–45.5)	702 (25.8)	25.8 (21.4–30.1)	
**Marital status**							
Never married	805 (28.0)	28.0 (26.2–29.8)	1817 (20.3)	20.3 (19.1–21.6)	142 (5.5)	5.5 (4.2–6.8)	<0.0001
Currently married	1712 (59.9)	59.9 (58.1–61.8)	6873 (72.4)	72.4 (71.2–73.7)	2395 (88.7)	88.7 (87.2–90.2)	
Formerly/ever married	347 (12.1)	12.1 (10.8–13.3)	668 (7.2)	7.2 (6.6–7.9)	178 (5.8)	5.8 (4.8–6.8)	
**Wealth quintile**							
Poorest	824 (22.8)	22.8 (19.4–26.2)	2283 (19.9)	19.9 (17.3–22.6)	239 (6.9)	6.9 (5.1–8.6)	<0.0001
2	692 (23.5)	23.5 (20.4–26.6)	2239 (22.0)	22.0 (19.9–24.1)	404 (12.6)	12.6 (10.4–14.8)	
3	622 (22.8)	22.8 (20.1–25.5)	2041 (21.9)	21.9 (19.9–23.9)	553 (18.4)	18.4 (15.4–21.3)	
4	418 (17.4)	17.4 (14.5–20.3)	1480 (18.7)	18.7 (16.5–20.9)	609 (23.9)	23.9 (21.1–26.7)	
Richest	308 (13.5)	13.5 (10.6–16.4)	1315 (17.5)	17.5 (14.8–20.2)	910 (38.3)	38.3 (33.8–42.8)	
**Ecological region**							
Mountain	235 (6.1)	6.1 (3.9–8.3)	795 (7.4)	7.4 (4.8–10.0)	143 (4.9)	4.9 (2.2–7.5)	<0.0001
Hill	1037 (30.9)	30.9 (26.0–35.7)	4369 (45.1)	45.1 (40.1–50.1)	1348 (51.5)	51.5 (45.1–57.9)	
Terai	1592 (63.1)	63.1 (58.2–67.9)	4194 (47.6)	47.6 (42.9–52.2)	1224 (43.6)	43.6 (37.4–49.8)	
**Provinces**							
Province 1	369 (15.3)	15.3 (12.6–18.0)	1363 (17.8)	17.8 (16.3–19.2)	481 (18.9)	18.9 (16.1–21.8)	<0.0001
Province 2	715 (32.7)	32.7 (29.1–36.2)	1452 (19.4)	19.4 (17.7–21.2)	317 (12.4)	12.4 (10.1–14.6)	
Province 3	217 (12.2)	12.2 (8.4–16.0)	1289 (20.8)	20.8 (17.4–24.2)	573 (32.8)	32.8 (27.2–38.5)	
Province 4	226 (6.0)	6.0 (4.9–7.1)	1223 (10.3)	10.3 (9.3–11.2)	529 (14.2)	14.2 (11.8–16.6)	
Province 5	443 (17.3)	17.3 (14.4–20.1)	1381 (16.4)	16.4 (15.0–17.8)	418 (15.3)	15.3 (12.5–18.0)	
Province 6	395 (5.8)	5.8 (4.8–6.9)	1306 (6.2)	6.2 (5.6–6.8)	203 (2.3)	2.3 (1.6–3.0)	
Province 7	499 (10.7)	10.7 (9.3–12.2)	1344 (9.1)	9.1 (8.2–10.0)	194 (4.1)	4.1 (2.2–6.0)	

### Prevalence of underweight and overweight and/or obesity by wealth quintiles and geography

**Figure 1:**
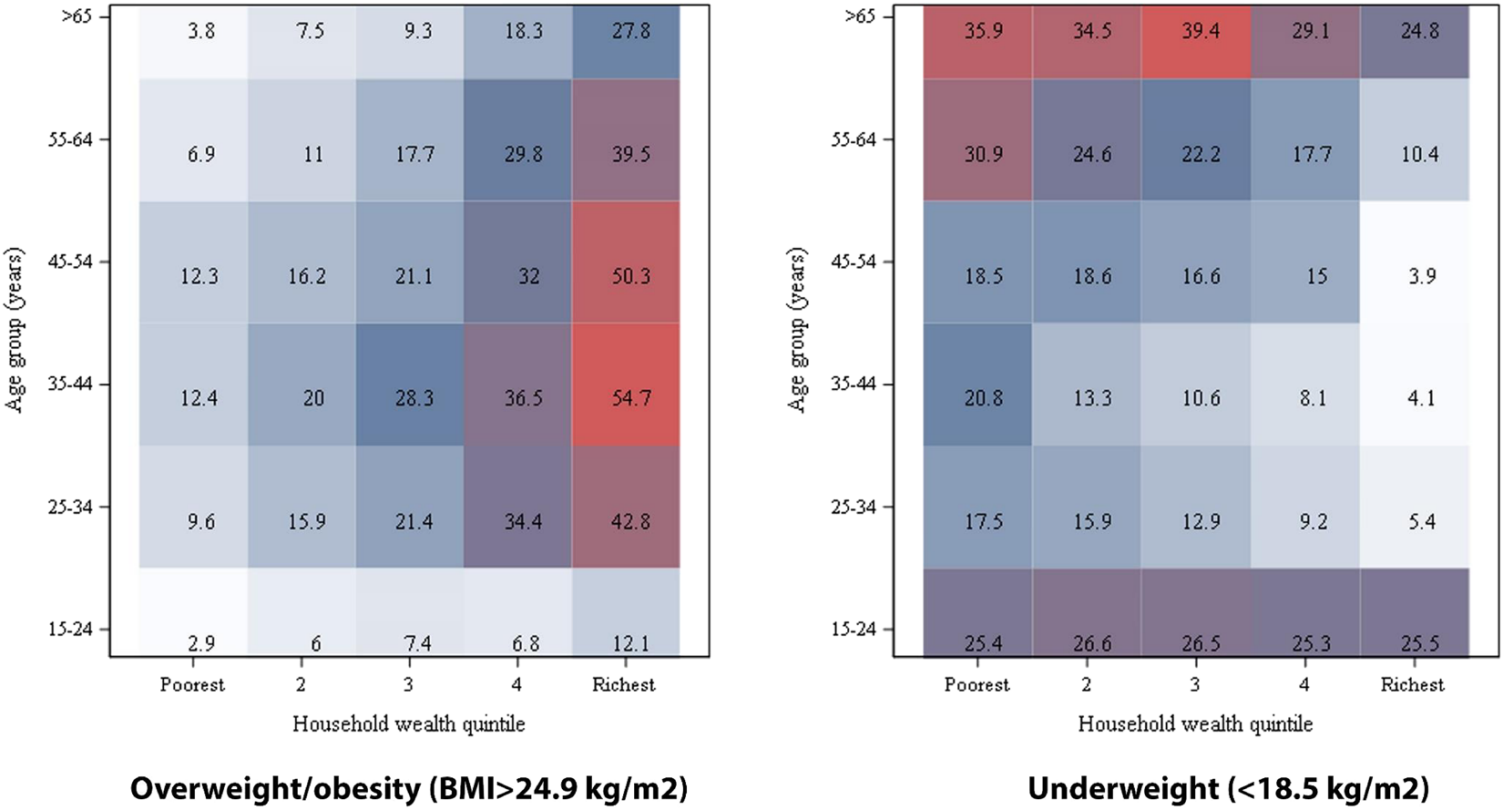
Heat map showing the prevalence of overweight/obeisty (BMI >24.9 kg/m^2^) and underweight (<18.5 kg/m^2^) by age groups and household wealth quintiles.

The heat map (Fig. 1, Supplementary Fig. 1) for the prevalence of overweight and/or obesity and underweight by age categories and wealth quintiles suggests that middle-aged adults (35–54 years) in the highest wealth quintile (quintile 5) are more vulnerable to overweight and/or obesity; the prevalence for the age category in the given wealth quintile was greater than 50% based on both global classification of BMI (BMI >24.9 kg/m2) and the classification for Asians (BMI >22.9 kg/m2). The heat map for undernutrition shows that older adults, aged 65 and above, in the lower- and mid-wealth quintiles (quintiles 1, 2, 3) were vulnerable to underweight, where more than one-third of the older adults in the given quintiles were underweight (Fig. 1, Supplementary Fig. 1).

**Figure 2:**
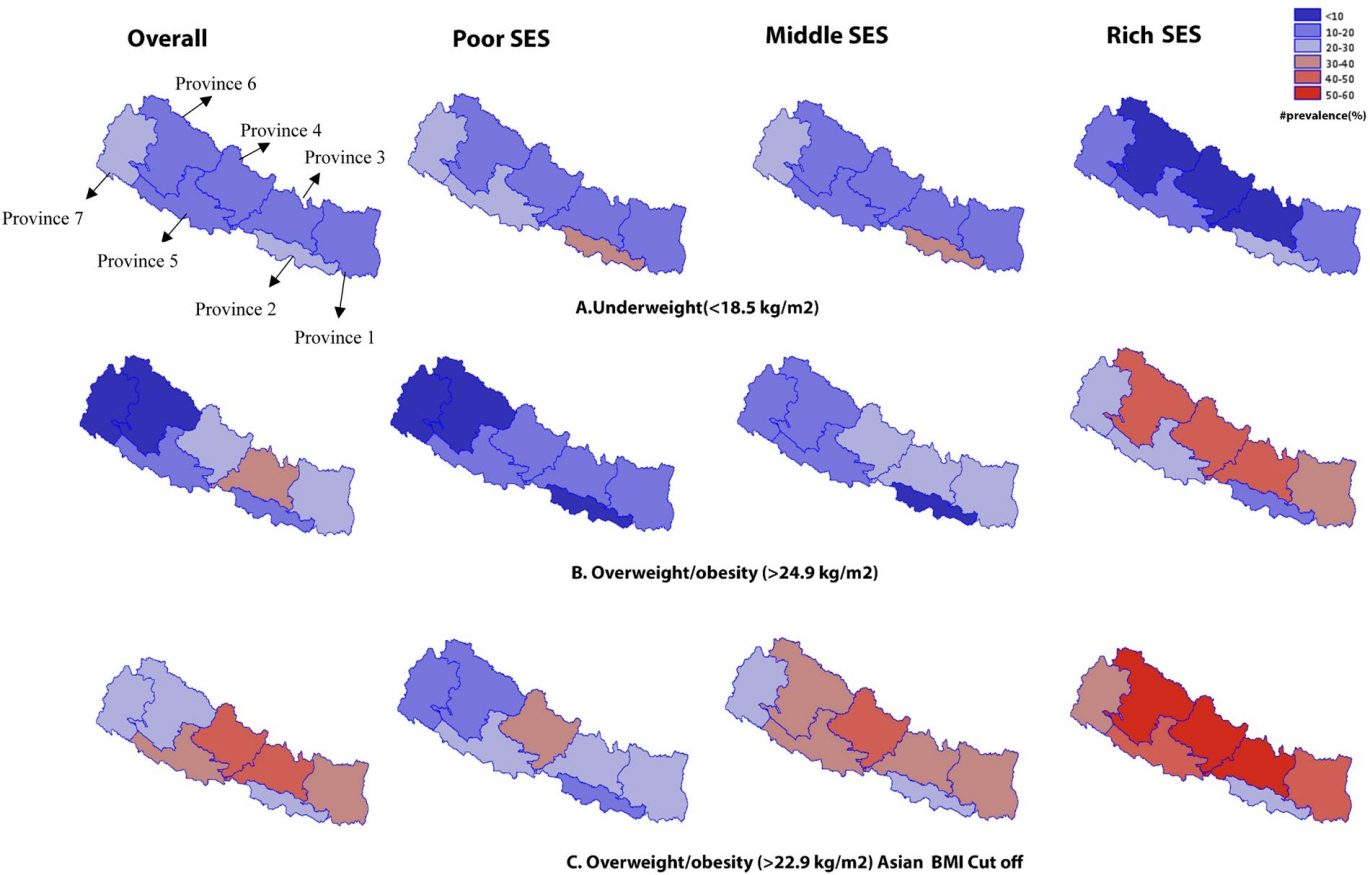
Overweight and/or obesity (>24.9 kg/m^2^), overweight and/or obesity (>22.9 kg/m^2^) and underweight (<18.5 kg/m^2^) by wealth status. Socio-economic status (SES) is defined using principal component analysis into quintiles: ‘poorest’ and ‘poor’ is merged as ‘Poor SES’, ‘richer’ and ‘richest’ is merged as ‘Rich SES’ whereas ‘middle’ remained as 'Middle SES'.

The geospatial analyses in Fig. 2 show the prevalence of overweight and/or obesity and underweight by wealth quintiles. The prevalence of underweight was higher among the residents of Province-2 who belonged to lower wealth quintiles (quintile 1, 2, and 3). Overweight and/or obesity was more prevalent (>40% by global cutoff and >50% by Asian cutoff) among the residents of Province-3, 4, and 6 who belonged to higher wealth quintiles (quintile 4 and 5) (Fig. 2).

**Figure 3:**
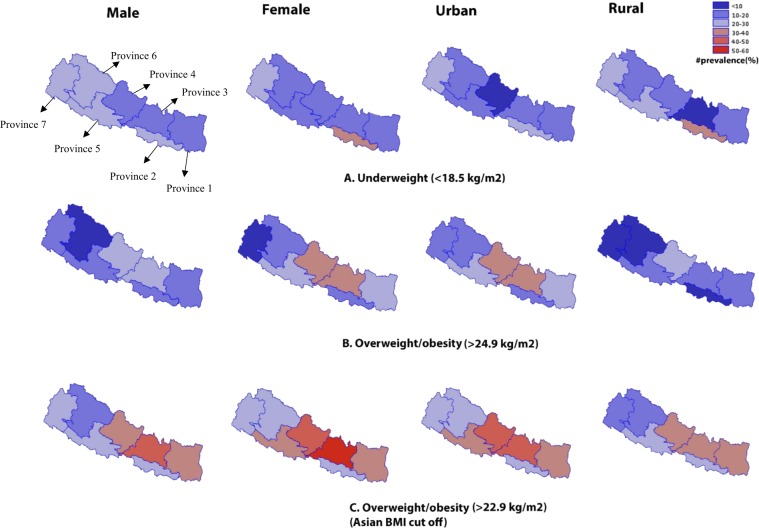
Overweight and/or obesity (>24.9 kg/m^2^), overweight and/or obesity (>22.9 kg/m^2^) and underweight (<18.5 kg/m^2^) by sex and residence.

The geospatial analyses presented in Fig. 3 shows the prevalence of overweight and/or obesity and underweight by gender and residence. Undernutrition, particularly among females and rural residents, is comparatively higher in Province-2; prevalence ranged between 30–40%. Overweight and/or obesity was higher (≥30%) in Provinces-3 and 4, specifically among females and urban residents (Fig. 3).

### Single-level multinomial regression

In the single-level multinomial analyses (Tables 2 and 3), to assess the association between participants’ weight and their socio-economic characteristics, participant’s socio-economic and locational characteristics were associated with their both overweight and/or obesity and underweight status (Tables 2 and 3). After adjusting for age, sex, education, marital status, wealth quintiles, residency, ecological region, and provinces, compared to participants with no or only preschool education, those with any level of formal education had higher odds of being overweight and/or obese, and lower odds of being underweight (Table 2). Compared to rural residents, urban residents had higher odds of being overweight and/or obese (OR:1.89, 95% CI: 1.62–2.20) or lower odds of being underweight (OR:0.81, 95% CI: 0.70–0.93). Provinces wise, compared to the Province 1, participants from the Province 2, and 7 were less likely to be overweight and/or obese and more likely to be underweight. Ecologically, residents of Terai region had lower odds of being overweight and/or obese (OR: 0.81, 95% CI: 0.67–0.98) or higher odds of being underweight (OR: 1.74, 95% CI: 1.33–2.27) (Table 2).

**Table 2:** Obesity/overweight status by socio-economic variables (referent: normal weight): a multinomial logistic regression analysis

Variable Overweight &/or obesity Education*	Unadjusted	Model 1	Model 2	Model 3 (final model)	Model 4
No education, preschool	ref	ref	ref	ref	ref
Primary	1.32 (1.14–1.53)	2.05 (1.75–2.41)	1.59 (1.35–1.87)	**1.41 (1.20–1.66)**	**1.70 (1.44–2.00)**
Secondary	1.25 (1.09–1.44)	3.00 (2.54–3.54)	1.68 (1.42–1.99)	**1.49 (1.26–1.76)**	**2.42 (2.04–2.87)**
Higher	1.60 (1.35–1.90)	3.94 (3.23–4.79)	1.69 (1.36–2.11)	**1.46 (1.17–1.82)**	**3.13 (2.53–3.88)**
*p-trend*	<0.0001	<0.0001	<0.0001	<0.0001	<0.0001
**Rurality**					
Rural	ref		ref	ref	ref
Urban	1.99 (1.68–2.35)	2.08 (1.74–2.48)	2.14 (1.81–2.52)	**1.89 (1.62–2.20)**	**1.59 (1.34–1.87)**
**Provinces**					
Province 1	ref			ref	ref
Province 2	0.60 (0.47–0.75)	0.57 (0.45–0.73)		**0.55 (0.44–0.68)**	**0.58 (0.45–0.76)**
Province 3	1.48 (1.17–1.87)	1.56 (1.22–2.00)		1.01 (0.81–1.26)	**1.58 (1.22–2.06)**
Province 4	1.30 (1.03–1.64)	1.35 (1.06–1.73)		1.24 (1.00–1.53)	**1.46 (1.14–1.88)**
Province 5	0.87 (0.68–1.12)	0.87 (0.67–1.12)		0.81 (0.64–1.01)	0.86 (0.66–1.10)
Province 6	0.35 (0.25–0.49)	0.34 (0.24–0.48)		**0.55 (0.41–0.74)**	**0.44 (0.31–0.62)**
Province 7	0.42 (0.27–0.67)	0.41 (0.25–0.66)		**0.48 (0.33–0.70)**	**0.45 (0.29–0.71)**
**Ecological region**					
Mountain	0.58 (0.40–0.84)	0.56 (0.38–0.84)		1.24 (0.86–1.79)	0.96 (0.65–1.40)
Hill	ref			ref	ref
Terai	0.80 (0.67–0.95)	0.77 (0.64–0.93)		0.81 (0.67–0.98)	1.25 (1.02–1.53)
**Underweight**					
Education*					
No education, preschool	ref	ref	ref	ref	ref
Primary	0.71 (0.63–0.82)	0.69 (0.60–0.80)	0.72 (0.62–0.84)	**0.85 (0.73–0.98)**	**0.81 (0.70–0.93)**
Secondary	0.70 (0.63–0.79)	0.54 (0.46–0.63)	0.52 (0.44–0.62)	**0.67 (0.56–0.79)**	**0.59 (0.50–0.70)**
Higher	0.42 (0.34–0.52)	0.33 (0.26–0.42)	0.33 (0.26–0.42)	**0.50 (0.39–0.65)**	**0.39 (0.31–0.49)**
*p-trend*	<0.0001	<0.0001	<0.0001	<0.0001	<0.0001
**Rurality**					
Rural	ref	ref	ref	ref	ref
Urban	0.81 (0.69–0.94)	0.82 (0.70–0.96)	0.85 (0.73–1.00)	**0.81 (0.70–0.93)**	0.91 (0.80–1.05)
**Provinces**					
Province 1	ref	ref		ref	ref
Province 2	1.96 (1.53–2.50)	2.03 (1.58–2.62)		**1.71 (1.30–2.24)**	**1.70 (1.29–2.23)**
Province 3	0.68 (0.49–0.95)	0.67 (0.48–0.95)		0.89 (0.64–1.23)	0.80 (0.56–1.14)
Province 4	0.68 (0.52–0.89)	0.65 (0.49–0.86)		0.78 (0.57–1.05)	0.77 (0.58–1.02)
Province 5	1.22 (0.93–1.60)	1.25 (0.94–1.65)		1.16 (0.89–1.52)	1.17 (0.90–1.51)
Province 6	1.10 (0.83–1.48)	1.15 (0.85–1.55)		1.29 (0.96–1.72)	**1.44 (1.06–1.96)**
Province 7	1.37 (1.06–1.79)	1.42 (1.08–1.87)		**1.39 (1.07–1.82)**	**1.47 (1.13–1.93)**
**Ecological region**					
Mountain	1.21 (0.93–1.57)	1.22 (0.92–1.61)		0.91 (0.70–1.17)	0.97 (0.75–1.27)
Hill	ref	ref		ref	ref
Terai	1.94 (1.70–2.20)	2.02 (1.76–2.30)		**1.74 (1.33–2.27)**	**1.42 (1.10–1.84)**
Model fitness	NA	NA	AIC = 25003.8 SBIC = 25262.7	AIC = 24292.8, SBIC = 24673.4	AIC = 25025.5, SBIC = 25345.2

Model 1: Four individual models adjusted for age and sex; Model 2: age, sex, education, marital status, wealth quintile and rurality; Model 3: age, sex, education, marital status, wealth quintile, rurality, ecological region, provinces (final -model); Model 4: model 3 without wealth quintile (which is used to compare the effect of wealth quintile on overall estimates).

Abbreviation: AIC: akaike information criterion, NA: not applicable, SBIC: schwarz bayesian information criterion.

*Wealth quintile with ordered exposure levels is entered as a linear predictor variable into the model.

**Table 3:** Obesity/overweight status by wealth quintile (referent: normal weight): a multinomial logistic regression analysis.

Variable Overweight &/or obesity Education	unadjusted	model 1	model 2	model 3
Poorest	ref	ref	ref	ref
2	1.67 (1.34–2.08)	1.74 (1.39–2.18)	1.73 (1.38–2.15)	**1.74 (1.40–2.16)**
3	2.44 (1.94–3.07)	2.54 (2.01–3.21)	2.41 (1.92–3.04)	**2.58 (2.03–3.29)**
4	3.72 (3.00–4.60)	4.11 (3.28–5.16)	3.82 (3.11–4.70)	**4.09 (3.30–5.06)**
Richest	6.36 (5.05–8.00)	7.00 (5.51–8.90)	6.66 (5.31–8.34)	**7.22 (5.71–9.13)**
p-trend*	<0.0001	<0.0001	<0.0001	<0.0001
**Underweight**				
Poorest	ref	ref	ref	ref
2	0.94 (0.80–1.10)	0.94 (0.79–1.11)	0.96 (0.81–1.13)	**0.82 (0.69–0.96)**
3	0.91 (0.77–1.08)	0.93 (0.78–1.11)	0.99 (0.83–1.18)	**0.69 (0.55–0.86)**
4	0.81 (0.66–1.00)	0.83 (0.67–1.03)	0.91 (0.74–1.13)	**0.61 (0.47–0.79)**
Richest	0.68 (0.55–0.84)	0.69 (0.55–0.85)	0.79 (0.63–0.98)	**0.50 (0.38–0.66)**
p-trend*	0.0007	0.0019	0.0835	<0.0001
Model fitness	NA	NA	AIC = 25003.8, SBIC = 25262.7	AIC = 24292.8, SBIC = 24673.4

Model 1: Individual model adjusted for age and sex, Model 2: model 1 plus education, marital status, and rurality, model 3: model 2 plus ecological region and provinces.

Abbreviation: AIC: akaike information criterion, NA: not applicable, SBIC: schwarz bayesian information criterion. *Wealth quintile with ordered exposure levels is entered as a linear predictor variable into the mode.

Participants with higher wealth quintiles were associated with higher odds of being overweight and/or obese and lower odds of being underweight (Table 3).

### Multinomial regression

The null model, containing no explanatory variables, i.e., only the outcome and a constant, was used to illustrate the total variance in overweight and/or obesity associated with individual and locational (and district) characteristics. The intercept and the Intra-class Correlation Coefficient (ICC) for provinces and districts were significantly different than zero in all the models, suggesting that the log-odds varied significantly between the provinces and the districts. For the null model, the ICC for level 2 and level 3 was 6.8 and 7.3, respectively. This means that about 15% of the variance in overweight and/or obesity is explained by population differences (6.8% due to the systematic differences between provinces and 7.3% due to districts) and more than 85% of the variance is attributable to individual differences. In the fully adjusted model 4, the ICC reduced to 4.1% intra-provincial correlation and 2.9% intra-district correlation suggesting that only a small variation in overweight and/or obesity was explained by random differences between the provinces and the districts.

The value of Akaike’s information criteria (AIC) and Schwarz Bayesian information criteria (SBIC) were reduced with the addition of covariates across the four models suggesting that the subsequent model improved over the previous model. The model 4 was the best fitting model as suggested by the smallest value of AIC and SBIC compared to the preceding model (Table 4). Compared to the null model, the proportional change in the variance of overweight and/or obesity, due to addition of age, sex, wealth quintiles, education, marital status, and residency in the model 4, was 39.7% across provinces and 60.3% across districts which indicated that 39.7% of the provincial variance and 60.3% of the variance by districts in the empty model was attributable to differences in age, sex, wealth quintiles, education, marital status, and residency.

**Table 4:** Multilevel logistic regression analysis for overweight &/or obesity (referent: normal weight) (N = 12,073).

Variables	model 1	model 2			model 3			model 4		
		OR	LCL	UCL	OR	LCL	UCL	OR	LCL	UCL
Intercept	**−1.52(0.18)**	**−2.612(0.20)**			**−2.93(0.21)**			**−3.525(0.19)**		
**Age(years)**										
15–25		ref			ref			ref		
25–35		4.29	3.67	5.01	4.147	3.5	4.86	2.81	2.35	3.35
35–45		5.83	4.97	6.83	5.872	5	6.9	4.05	3.35	4.90
45–55		5.05	4.27	5.98	5.136	4.3	6.1	3.85	3.13	4.74
55–65		3.74	3.10	4.50	3.847	3.2	4.65	3.11	2.47	3.92
>65		2.44	1.97	3.02	2.571	2.1	3.2	2.32	1.77	3.04
**Sex**										
Male		0.70	0.64	0.77	0.681	0.6	0.75	0.62	0.55	0.69
Female										
**Education**										
No education, preschool					ref			ref		
Primary								1.45	1.25	1.68
Secondary								1.55	1.33	1.79
Higher								1.56	1.30	1.88
**Marital status**										
Formerly/ever married								0.82	0.67	1.00
Never married								0.34	0.27	0.42
Currently married								ref		
**Residency**										
Urban								1.66	1.45	1.91
Rural								ref		
**Wealth quintile**										
Poorest					ref			ref		
2					2.478	2.1	2.98	2.37	1.97	2.86
3					1.614	1.3	1.94	1.61	1.34	1.94
4					3.904	3.2	4.74	3.67	3.02	4.46
Richest					6.263	5.2	7.6	6.08	4.97	7.45
**Error variance**										
Level 2 intercept*	0.24(0.15)	0.23(0.14)			0.16(0.10)				0.14(0.084)	
Level 3 intercept*	0.26(0.06)	0.38(0.069)			0.19(0.04)				0.10(0.029)	
*ICC-level 2*	6.80	6.50			4.60				4.10	
*ICC-level 3*	7.30	10.40			5.50				2.90	
*PCV-level 2*		4.4%			32.4%				39.7%	
*PCV-level 3*		42.5%			24.7%				60.3%	
*AIC*	12306.2	11491.7			11060.6				10868.3	
SBIC	12306.0	11473.7			11034.6				10830.3	

Model 1: empty model, model 2: adjusted for age and sex, model 3: model 2 plus wealth quintile, and model 4: model 3 plus education, marital status, and residency. The level two intercepts are for provinces and level 3 are for the districts. Abbreviation: AIC: akaike information criterion, LCL: lower conflidence limit, SBIC: schwarz bayesian information criterion, UCL: upper confidence limit.

In the best fitting multilevel model, older ages compared to the youngest (15–25 years), female sex, any level of school education compared to illiterate or with only preschool education, residing in urban areas, and higher wealth quintiles compared to the first quintile was associated with higher odds of being overweight and/or obese. Never married participants were less likely to be overweight and/or obese than those married (OR: 0.34, 95% CI: 0.27–0.42) (Table 4).

In the multilevel analyses for underweight, the intra-provincial correlation and intra-district correlation for underweight was 3.5% and 2.1%, respectively which slightly increased to 4.6% and 3.8%, respectively in the final model (model 4). Thus, only a small variation in underweight was explained by random differences between the provinces and the districts, and the individual differences explained most of the variance. As suggested by the smaller value of AIC and SBIC, model 4 was the best fitting model. Compared to the null model, there was a decrease in the proportion of provincial (31.4%) and district level (81.0%) variance with the addition of individual-level predictors in the final model.

The best fitting multilevel model for underweight suggests that compared to the youngest age group (15–25 years), adults in the middle ages (25–55 years) had lower odds of being underweight but the older adults (>65 years) had higher odds of being underweight (OR:1.85, 95% CI: 1.50–2.29). Compared to illiterate or with only preschool education, participants with any level of school education were less likely to be underweight. Being married appeared to be protective against underweight compared to being unmarried (OR:2.30, 95% CI:1.99–2.67), and previously married (OR:1.20, 95% CI:1.02–1.42) participants were more likely to be underweight than their married counterparts. Urban residents were less likely to be underweight compared to rural (OR:0.87, 95% CI: 0.76–0.99) but the statistical significance was borderline. The financial privilege was protective against underweight, as participants in the higher wealth quintiles had lower odds of being underweight compared to the first quintile (Table 5).

**Table 5:** Multilevel logistic regression analysis for underweight (referent: normal weight) (N = 12,222).

Variables	model 1	model 2			model 3			model 4		
		OR	LCL	UCL	OR	LCL	UCL	OR	LCL	UCL
Intercept	−1.28(0.14)	−1.03(0.15)			−1.04(0.17)			−0.82(0.18)		
Age (years)										
15–25		ref			ref			ref		
25–35		0.45	0.40	0.52	0.448	0.4	0.51	0.63	0.53	0.74
35–45		0.48	0.41	0.55	0.46	0.4	0.53	0.60	0.50	0.73
45–55		0.59	0.50	0.68	0.566	0.5	0.66	0.71	0.58	0.86
55–65		1.05	0.91	1.21	1.005	0.9	1.16	1.19	0.98	1.46
>65		1.74	1.51	1.99	1.68	1.5	1.93	1.85	1.50	2.29
**Sex**										
Male		0.89	0.81	0.97	0.899	0.8	0.98	0.92	0.83	1.01
Female		ref			ref					
**Education**										
No education, preschool										
Primary								0.83	0.72	0.96
Secondary								0.69	0.59	0.80
Higher								0.52	0.42	0.63
**Marital status**										
Formerly/ever married								1.20	1.02	1.42
Never married								2.30	1.99	2.67
Currently married								ref		
**Residency**										
Urban								0.87	0.76	0.99
Rural										
**Wealth quintile**										
Poorest					ref			ref		
2					0.672	0.6	0.78	0.70	0.61	0.81
3					0.784	0.7	0.9	0.80	0.70	0.92
4					0.562	0.5	0.67	0.60	0.51	0.72
Richest					0.448	0.4	0.54	0.48	0.39	0.59
**Error variance**										
Level 2 intercept*	0.12(0.07)	0.14(0.08)			0.16(0.09)				0.16(0.09)	
Level 3 intercept*	0.07(0.02)	0.10(0.02)			0.14(0.03)				0.13(0.03)	
*ICC-level 2*	3.50	3.40			4.60				4.60	
*ICC-level 3*	2.10	2.90			4.10				3.80	
*PCV-level 2*		2.9%			−31.4%				−31.4%	
*PCV-level 3*		−38.1%			−95.2%				−81.0%	
*AIC*	13053.4	12640.9			12563.8				12403.0	
*SBIC*	13053.2	12622.9			12537.7				12364.9	

Model 1: empty model, model 2: adjusted for age and sex, model 3: model 2 plus wealth quintile, and model 4: model 3 plus education, marital status, and residency. The level two intercepts are for provinces and level 3 are for the districts. Abbreviation: AIC: akaike information criterion, LCL: lower confidence limit, SBIC: schwarz bayesian information criterion, UCL: upper confidence limit.

### Socio-economic inequalities in weight status: Lorenz curves analyses

**Figure 4:**
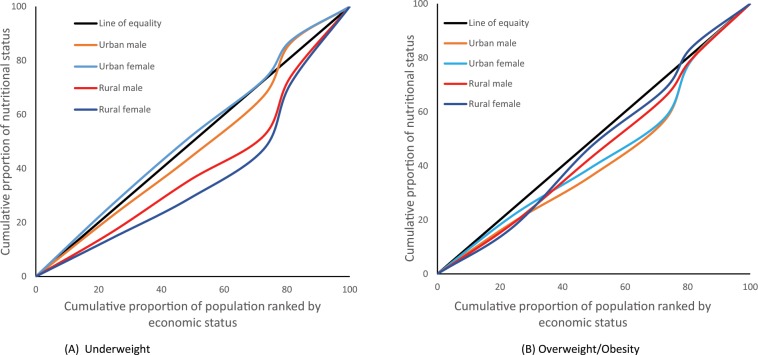
Lorenz curves showing the gradient in overweight and/or obesity (>24.9 kg/m^2^) and underweight (<18.5 kg/m^2^).

Analyses using the Lorenz curves (Fig. 4, Supplementary Fig. 2), used for understanding inequality between richer and poorer and disaggregated by sex and residency, further confirmed the earlier findings in multivariable regression that showed a positive association between weight status and wealth quintiles. The Lorenz curves for the prevalence of underweight (Fig. 4A) shows that underweight status was most heavily concentrated among poor males and females in the urban area and among rich males and females in the rural area. The concentration curve graph shows that, urban female at the bottom 20% were more vulnerable as nearly 39% were underweight and at the top 20% nearly 19% were underweight (Fig. 4A).

Overweight and/or obesity prevalence was concentrated among the rich as shown by all the curves below the line of equality. Particularly, urban females at the highest wealth quintile were more vulnerable as 49% were overweight and/or obese (Fig. 4B). This is not very different from the overweight and/or obesity gradient based on Asian BMI cut off (Supplementary Fig. 2).

## Discussion

### Summary of evidence

This study reports the prevalence of underweight and overweight and/or obesity, estimated by measures of height and weight, in a nationally representative sample of Nepalese adults. The findings of this study provide evidence of the dual burden of underweight and overweight and/or obesity, demonstrating that this is an urgent priority for the health of Nepal. Our study demonstrates that the prevalence of overweight and/or obesity was higher in younger adults and undernutrition was higher among the older adults. Based on wealth indicators, individuals from richer households were least likely to be underweight and more likely to overweight and/or obese. The multilevel analyses suggest that only a small variation in overweight and/or obesity and underweight can be explained by random differences between the provinces and the districts, and the individual differences explained most of the variance.

### Comparison to existing studies

Though the prevalence and factors affecting underweight and overweight and/or obesity from NDHS 2016 were reported in earlier studies^13,14^, none of these studies examined the geographical and socio-economic variation of weight status with a detailed spatial mapping of BMI status. And, both studies have some methodological limitations. For example, Al Kibria^13^ used the same weight for the underweight vs normal weight comparison, and overweight and/or obesity vs normal weight comparisons, though they were analysed separately using multivariable logistic regression analysis. Though, a detailed account of modeling process was lacking in these studies^13,14^. These shortcomings have been addressed in our study. We used a hierarchical multi-nominal regression analysis design where normal weight used as referent category and compared against underweight and overweight and/or obesity under the same model. Further, we used the multilevel analysis to examine the population level differences in BMI status, and Lorenz curves to study socio-economic inequalities in BMI status. Both multi-nominal and multilevel analysis explored the same set of predictor variables and the findings have high congruence between two modeling approaches. Multilevel analysis is accounted for the presence of data hierarchies and the random residual components at each level in the study^17^. It is superior to the traditional logistic regression modeling^17^, however, we did not aim to compare the results between single level and multi-level analysis. Our study focuses on describing the drivers of nutritional transition, and their policy implications than the effect sizes of estimates.

### Dual burden of underweight and overweight and/or obesity

A notable proportion of participants were found to be underweight. The finding was not surprising given that historically undernutrition has been a significant public health problem in Nepal^18^. Underweight status appeared to be a major concern among the older adults in our study which is in line with previous evidence that the nutritional well-being of older adults is particularly neglected in Nepal^19,20^.

On the other hand, recently overweight and/or obesity has emerged as a notable public health concern. It is particularly of concern among young adults, given that being obese increases the vulnerability to sequelae of cardio-metabolic diseases. The national STEPS survey on non-communicable diseases risk factors, conducted in 2013, also provides evidence that Nepalese adults are becoming overweight and/or obese (obesity: 21%)^9^. Two aspects of Nepalese society, the traditional norms of being obese and rapid urbanization, may be attributed to the rise in obesity. Being overweight is culturally desirable in Nepalese society and is even used as a proxy of family’s ability to afford food as well as a measure of their relative wealth status^21^. This traditional phenomenon has been further complicated by the rapid urbanization which has increased the accessibility and affordability of energy-rich, sugary commercialized foods, and simultaneously, a more sedentary lifestyle^22,23,24^.

### Socio economic status (SES) and nutritional status

The relationship between weight status and SES was expected, yet interesting. We found a high prevalence of overweight and/or obesity among wealthier individuals, measured in terms of wealth index, and a high prevalence of underweight among poorer individuals. This is not unexpected, and consistent with other data from Nepal and globally^15,25^. The poorest quintile were two times more likely to be underweight than the richest quintile, whereas the richest quintile were 7.2 times more likely to be overweight and/or obese than the poorest quintile. However, the disparity in underweight across socio-economic groups is much more evident within urban areas compared to rural areas although there was no statistically significant association of residence with underweight. This large disparity between rich and poor subgroups in Nepal might be associated with ongoing rapid urbanization that will further widen income and social inequities. These findings corroborate to those reported by previous studies conducted in other LMICs^5,15,26^.

A higher burden of underweight status among the lower socio economic group in urban regions could be attributable to rapid growth of cities along with growth of urban poor^27^. Globally, South Asia has the largest proportion of the population living in slums and is urbanizing at the fastest rate^28^. The people living in these slums are often beyond the remit of public services and are forced to endure disparities in essential health services and health and nutrition indicators^29^. Policymakers cannot ignore the sheer scale and uncontrollable urban growth and as more and more of the populations in LMICs become urbanized, so the need for special attention for this population subgroup is growing^30^. In addition, the urbanization also incites inflation which makes consumption of fruits and vegetables expensive and increasingly difficult for urban poor. Thus the consumption of unhealthy foods along with the transition to sedentary occupations from subsistence agriculture might be the reasons behind the shift in the burden of obesity to lower-SES groups in LMICs^31^. With rapid urbanization, the urban poor subgroup in Nepal is also at risk for double burden of undernutrition and overweight and/or obesity.

### Geographic drivers

In the geospatial analyses, underweight status was more prevalent among the residents of Province 2, Province 6 and Province 7 whereas overweight and/or obesity was more prevalent among the residents of Province 3 and 4. Overweight and/or obesity is more prevalent in provinces and districts with higher affluence and underweight in less affluent provinces. For example, Province 1 and 3, with human development index (HDI) of 0.507 and 0.506, are the most developed provinces in terms of HDI as well as other indicators of economic growth, followed by Province 4 (HDI: 0.493). At the lower end, Province 6 (HDI: 0.39), Province 7 (HDI: 0.416), and Province 2 (HDI: 0.422) have the lowest HDI, with the majority of the population residing in the village and semi-urban area and engaged in agrarian activities as a primary source of income. When comparing the districts, the top three districts in terms of HDI are from Province-3: Kathmandu (HDI: 0.632), Lalitpur (0.601), Kaski (0.576), and the bottom three are from Province 7: Bajura (0.364), Bajhang (0.365), and Kalikot (0.374)^32^. At the individual level—underweight showed a negative trend in dose-response manner with household SES and overweight showed a positive trend. Low SES groups from low HDI districts and high SES groups from high HDI districts are particularly vulnerable to poor nutritional status and needs vigilant attention. In line with our findings, geographic disparitities in overweight and/or obeseity has been reported in previous studies from Nigeria and neighbouring India where overweight and/or obesity was more prevalent in Southern India ^33^ and south-eastern Nigeria^34^ compared to other regions of these countries.

Nepal is in a transitional phase and has recently transformed into a federal setup with seven administrative provinces. The several decades of political instability and ten years of armed conflict that ended in 2006, plunged Nepal into poverty. The situation is particularly grave for the Province 2, Province 6 and Province 7 with approximately half of their population being poor^35^. Though Province 2, the plains of Nepal has been the major region for crop production, food insecurity in this province is on the rise due to annual floods, crop failures and feudalistic distribution of land in agriculture. The difficult terrain in Province 6 and Province 7, along with poor crop harvest and poor income from agriculture and livestock has been the major reasons behind highest poverty incidence in these provinces. Other reasons are out-migration of youth into Persian Gulf countries and in-migration of richer households from rural to urban areas leaving the poorer behind as they are less likely to migrate because of lack of affordability^36^. Along with that, these provinces were the worst affected region during armed conflict. This is also reflected in higher underweight prevalence in these provinces and remains a challenge for Nepal.

### Strengths and limitations

Our study adds to the growing literature surrounding nutritional status in Nepal. To date, nationally representative data are limited in Nepal, and the prevalence, estimated in the earlier studies, are based on small sample sizes and among a homogenized population in a limited geography. This study is based on a large nationally representative sample consisting of both urban and rural populations in Nepal. This is the first study exploring the socio-economic and geographical disparities in the prevalence of underweight and overweight and/or obesity in the Nepalese context. Further, we defined overweight and/or obesity based on two different criteria, the global criteria and the criteria recommended for Asians. Our geospatial analyses may be helpful to the new provincial governments, especially in the context of recent federalization of the country, to design the targeted interventions based on local needs.

Our study also has several limitations. This study is cross-sectional in nature, and thus limits any causal inferences. Likewise, BMI and SES were measured only at one point, but such measurements are likely to change over time. Given the methods of our data sample, we were unable to assess the impact of such changes at different stages of the life course. Additionally, the lack of information regarding dietary habits, alcohol intake, or physical activity in the NDHS database hindered our ability to scrutinize some important determinants of nutritional status and the possibility of residual confounding cannot be ruled out. The geospatial mapping was entirely limited to data-visualization, and further studies needs to be carried out to identify the area-level hotspots of endemic underweight and overweight and/or obesity in Nepal. Despite the limitations, our study provides new insights into socio-economic and geographic determinants of weight status in Nepal.

## Conclusion

This study reaffirmed the earlier evidence that the dual burden of underweight and overweight and/or obesity is a significant public health problem in Nepal. The BMI status varied by indicators of socio-economic well-being which further confirms the role of the household’s economic well-being in the current epidemiological transition. Overweight and/or obesity among the younger adults and undernutrition among the elderly should receive special attention.

## Methods

### Data sources

This study is based on a secondary analysis of the 2016 NDHS, a cross-sectional and nationally representative data. The 2016 NDHS uses multi-stage (two stages in rural areas and three stages in urban areas) stratified cluster sampling technique. A total of 383 primary sampling units (PSU) or clusters were selected nationally, and 30 households were selected from each of these PSU with an equal probability proportion to size criterion yielding a final sample size of 11,040 households. From these households, 14,937 individuals, including 6,172 males and 8,765 females, provided height and weight measurements. Data includes individuals from all the 77 districts in the country, across all seven provinces. The full report on the 2016 NDHS study design and sampling technique is available elsewhere^37,38^. Ethical approval for 2016 NDHS was obtained from Nepal Health Research (NHRC) – Ethical Review Board and ICF Institutional Review Board.

### Anthropometry measurements

The trained field research staffs measured the height and weight of the participants. BMI was calculated as weight in kilograms divided by the square of the height in meters (kg/m^2^). We followed two standard classifications of BMI. First, we used the global standard to categories BMI as underweight (<18.5 kg/m^2^), normal (18.5–24.9 kg/m^2^), overweight (25–29.9 kg/m^2^), and obese (>30 kg/m^2^); the latter two categories were combined to form a single category overweight and/or obese (≥25 kg/m^2^)^39^. Further, to provide a comparative context in the figures (heat map, geospatial maps, and Lorenz curves), we also calculated and presented the prevalence of overweight and/or obese (≥23 kg/m^2^) following the recommendation by WHO’s expert consultation on BMI classifications for Asians^40^.

### Explanatory variables

The explanatory variables that were selected for analysis can be grouped into socio-demographic, and geographic variables. We considered age of the participants, sex, educational status and wealth index under socio-demographic variables. Educational status was grouped into four categories: no formal schooling, primary schooling, secondary schooling and those with higher education (includes high school, college/university, postgraduate and above). The principal component analysis was used to determine wealth index which included information on number and kinds of consumer goods such as bicycle or car owned by household and housing characteristics (such as the source of drinking water, availability of toilet facilities^41^. Place of residence (rural/urban), Province and Ecological zone (Mountain/Hill/Terai) were included under geographic variables. The province was categorized into seven administrative divisions, according to the current administrative structure of Nepal, and compared against Province-1. Province 1 was chosen a reference category as it is most advantaged in political and economic means Nepal^42^. Wealth quintiles were computed by dividing the distribution into five equal categories, each comprising 20% of the population.

### Data analysis

#### Multivariable analysis

Analyses were adjusted for cluster and sample weight to ensure representativeness of provinces and to the urban and rural areas. Rao-Scott chi-square tests were used to assess the association between weight status and the explanatory variables in the bivariate analyses. Univariate multinomial logistic regression was performed to assess the association of weight status with various socio-demographic factors, as listed in Table 1, with normal weight as the reference category. We built three hierarchical models under multinomial analyses. Model 1 was adjusted for age and sex; model 2 was adjusted for variables from the model 1 plus education (E), marital status (M), wealth quintiles (SES), and residency (R). Model 3 was adjusted by adding ecological region (Ec) and provinces (P) to the model 2. Model 4 contained all the variables from the model 3 excluding the wealth quintiles. The fully adjusted model is summarized as:

BMI_ij_ = j, if alternative j is selected. Where BMI is a categorical outcome variable with possible values from i to j^43^.

$${{\rm{BMI}}}_{{\rm{ij}}}={{\rm{\beta }}}_{{\rm{o}}}+{{\rm{\beta }}{\rm{age}}}_{{\rm{ij}}}+{{\rm{\beta }}{\rm{sex}}}_{{\rm{ij}}}+{{\rm{\beta }}{\rm{SES}}}_{{\rm{ij}}}+{{\rm{\beta }}{\rm{E}}}_{{\rm{ij}}}+{{\rm{\beta }}{\rm{M}}}_{{\rm{ij}}}+{{\rm{\beta }}{\rm{R}}}_{{\rm{ij}}}+{{\rm{\beta }}{\rm{Ec}}}_{{\rm{ij}}}+{{\rm{\beta }}{\rm{P}}}_{{\rm{ij}}}+{{\rm{e}}}_{{\rm{ij}}}$$

Trend tests were performed for wealth quintiles and education (both used as an ordinal variable in the models) to evaluate the linear trends. The multinomial analysis accounting for complex survey design of NDHS 2016 was conducted based on earlier established procedure in SAS 9.4^44^.

#### Multilevel analysis

The hierarchical nature of NDHS data allowed us to perform a hierarchical generalized linear modeling using PROC GLIMMIX procedure in SAS 9.4^45^. Such dataset is ideal for exploring socio-economic and geographic differences in BMI status at individual and population level. We performed three-level analyses with individuals at level 1 (*a*), districts at level 2 (*b*), and provinces at level 3 (*c*), using the same set of predictor variable used in multivariable analysis. In the multi-level analyses, the model 1 is an empty model. The detailed model building process for three-level multilevel analysis is described by Kim *et al*.^46^ using BMI as a continuous outcome variable, and is adapted for categorical BMI as a categorical variable. The study found that the residuals are independently and identically distributed at the individual, and population level in NDHS 2016^46^. We further tested the model fit and influence statistics for the hierarchical data (Supplementary Material 1).

$${{\rm{BMI}}}_{{\rm{abc}}}={{\rm{\beta }}}_{0}+{({\rm{e}}}_{0{\rm{abc}}}+{{\rm{u}}}_{0{\rm{bc}}}+{{\rm{v}}}_{0{\rm{c}}})$$

In the equation above, B_0_ is the average BMI across all the provinces and the bracketed terms are the random effect terms association with individual, districts and provinces.V_0C_, u_0bc_, e_0abc_ are the residual associated at the province, district and individual level respectively.

The model 2 was adjusted for age and sex.

$${{\rm{BMI}}}_{{\rm{abc}}}={{\rm{\beta }}}_{{\rm{o}}}+{{\rm{\beta }}{\rm{age}}}_{{\rm{abc}}}+{{\rm{\beta }}{\rm{sex}}}_{{\rm{abc}}}+{({\rm{e}}}_{0{\rm{abc}}}+{{\rm{u}}}_{0{\rm{bc}}}+{{\rm{v}}}_{0{\rm{c}}})$$

The model 3 was adjusted for model 2 variables plus wealth quintiles.

$${{\rm{BMI}}}_{{\rm{abc}}}={{\rm{\beta }}}_{{\rm{o}}}+{{\rm{\beta }}{\rm{age}}}_{{\rm{abc}}}+{{\rm{\beta }}{\rm{sex}}}_{{\rm{abc}}}+{{\rm{\beta }}{\rm{SES}}}_{{\rm{abc}}}+{({\rm{e}}}_{0{\rm{abc}}}+{{\rm{u}}}_{0{\rm{bc}}}+{{\rm{v}}}_{0{\rm{c}}})$$

The model 4 contained the model 3 variables plus education, marital status, and residency.

$${{\rm{BMI}}}_{{\rm{abc}}}={{\rm{\beta }}}_{{\rm{o}}}+{{\rm{\beta }}{\rm{age}}}_{{\rm{abc}}}+{{\rm{\beta }}{\rm{sex}}}_{{\rm{abc}}}+{{\rm{\beta }}{\rm{SES}}}_{{\rm{abc}}}+{{\rm{\beta }}{\rm{E}}}_{{\rm{abc}}}+{{\rm{\beta }}{\rm{M}}}_{{\rm{abc}}}+{{\rm{\beta }}{\rm{R}}}_{{\rm{abc}}}+{({\rm{e}}}_{0{\rm{abc}}}+{{\rm{u}}}_{0{\rm{bc}}}+{{\rm{v}}}_{0{\rm{c}}})$$

The intraclass correlation coefficient (ICC) (a measure of the amount of variation in outcome due to a given level) is calculated using the formula below where ${\tau }_{00}$ is the covariance parameter estimate.

$${\rm{ICC}}=\frac{{\tau }_{00}}{{\tau }_{00}+3.29}$$

The proportional change in variance (a measure of change in the area level variance between the empty model and a given successive model) was calculated based on the earlier established procedure in SAS v9.4^45^.

$$(\frac{{\sigma }_{empty\,model-{\sigma }_{fully\,adjusted}^{2}}^{2}}{{\sigma }_{empty\,model}^{2}})\ast 100$$

The value of AIC and SBIC were used as model fit statistics.

#### Geographic analysis and Lorenz curves

The geospatial analyses of provinces of Nepal were performed in SAS v9.4 using GMAP procedure^47^. The Lorenz curve assessed the socio-economic inequality in underweight and overweight and/or obesity among rural and urban residents. This curve has been used to assess the inequality gradient related to socio-economic status (SES) in various health indicators^48–50^. We plotted the cumulative percentage of the sample, ranked by SES on X-axis and the corresponding cumulative percentage of weight status, by sex and urban-rural residence, was plotted on the Y-axis. A curve above the line of equality indicates a greater concentration of the outcome among the poor and a curve below the line indicates a greater concentration of outcome among the rich.

### Ethical approval and consent to participate

The 2016 Nepal Demographic and Health Survey protocol received ethical approval by the Nepal Health Research (NHRC) – Ethical Review Board and ICF Institutional Review Board. The survey was conducted in accordance with relevant guidelines/regulations. An informed written consent was obtained from all the participants.

## Supplementary information

Supplementary Figures

Supplementary material 1
