# Supplementary material for: Application of single-level and multi-level modeling approach to examine geographic and socioeconomic variation in underweight, overweight and obesity in Nepal: findings from NDHS 2016
Source: Sci Rep. 2020 Feb 12;10:2406. doi: 10.1038/s41598-019-56318-w (PMC7016110; doi:10.1038/s41598-019-56318-w)

Application of single-level and multi-level modeling approach to examine the geographic and socioeconomic variation in underweight, overweight and obesity in Nepal: findings from NDHS 2016

Authors: Nipun Shrestha[1*], Shiva Raj Mishra[2*], Saruna Ghimire[3], Bishal Gyawali[4], Pranil Man Singh Pradhan[5], Dan Schwarz[6, 7, 8, 9]

1. Institute for Health and Sport (IHeS), Victoria University, Melbourne, Australia
2. Nepal Development Society, Chitwan, Nepal
3. Department of Sociology and Gerontology, Miami University, Oxford, OH
4. Department of Public Health, Aarhus University, Bartholins Allé 2, 8000 Aarhus C, Denmark.
5. Department of Community Medicine and Public Health, Institute of Medicine, Tribhuvan university
6. Nyaya Health Nepal, Kathmandu, Nepal
7. Ariadne Labs, Harvard T.H. Chan School of Public Health and Brigham and Women's Hospital, Boston, MA, USA
8. Division of Global Health Equity, Department of Medicine, Brigham and Women's Hospital, Boston, MA, USA
9. Department of Medicine, Harvard Medical School, Boston, MA, USA

* Nipun Shrestha and Shiva Raj Mishra contributed equally to the manuscript

Corresponding author
Shiva Raj Mishra
Nepal Development Society, Chitwan, Nepal
Email: shivarajmishra@gmail.com

Keywords: Obesity, Underweight, Nepal, Urban poor

Supplementary Figure 1. Heat map showing the prevalence of overweight/obesity (BMI>22.9 kg/m^2) by age groups and household wealth quintiles.

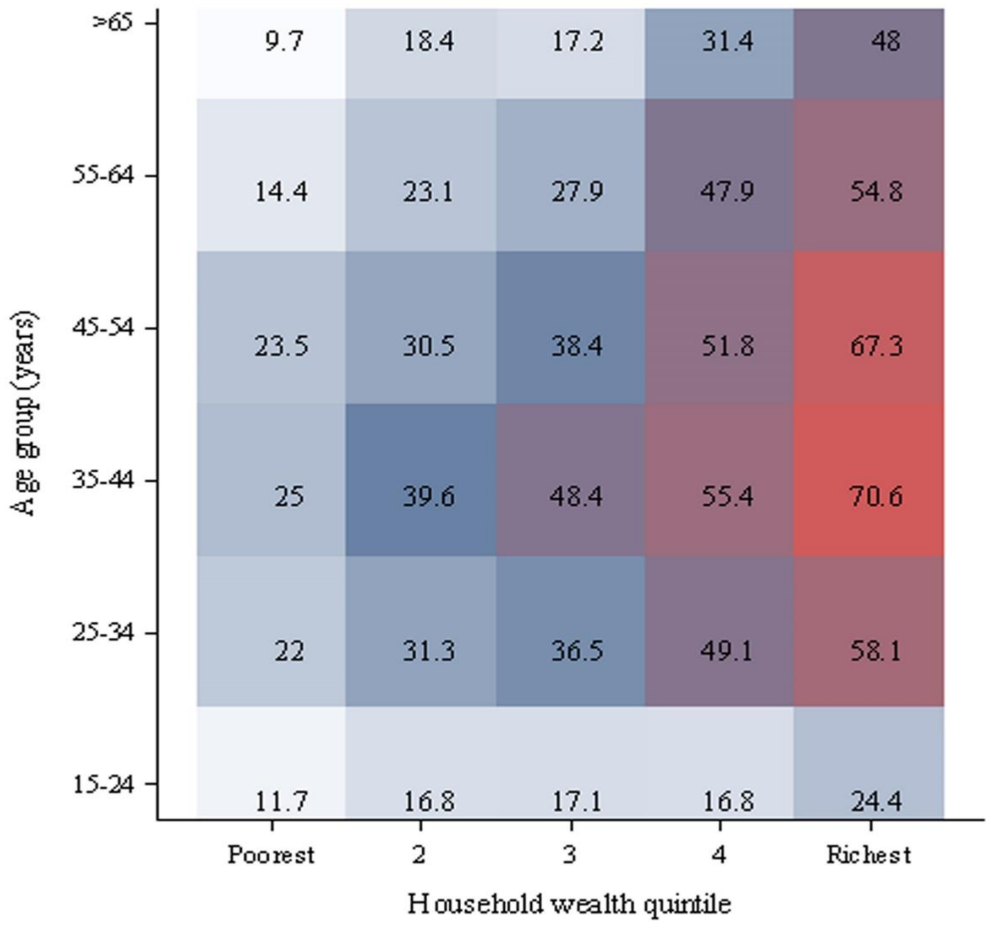

Overweight/obesity (>22.9 kg/m2)

Supplementary Figure 2: Concentration curve for Asian cut-offs overweight/obesity disaggregated by residence

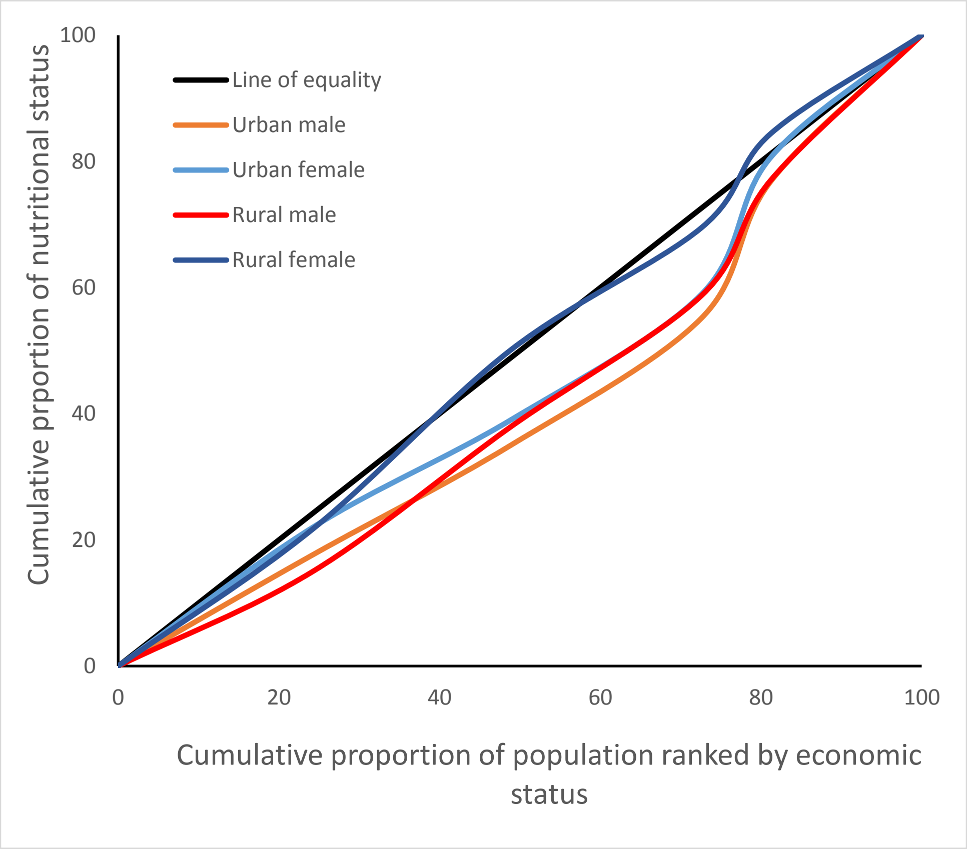

Supplement: Supplementary file 1 — Supplementary Figures [file 41598_2019_56318_MOESM1_ESM.pdf]
