# Supplementary material for: Application of single-level and multi-level modeling approach to examine geographic and socioeconomic variation in underweight, overweight and obesity in Nepal: findings from NDHS 2016
Source: Sci Rep. 2020 Feb 12;10:2406. doi: 10.1038/s41598-019-56318-w (PMC7016110; doi:10.1038/s41598-019-56318-w)
Supplement: Supplementary file 2 — Supplementary material 1 [file 41598_2019_56318_MOESM2_ESM.docx]

**Supplementary material 1**

Table 1: ROC Association Statistics

| **ROC Association Statistics** | | | | | | | |
| --- | --- | --- | --- | --- | --- | --- | --- |
| ROC Model | Mann-Whitney | | | | Somers' D | Gamma | Tau-a |
|  | Area | Standard Error | 95% Wald Confidence Limits | |  |  |  |
| Overweight/obesity vs normal weight (Figure 1) | | | | | | | |
| Empty model | 0.5000 | 0 | 0.5000 | 0.5000 | 0 | . | 0 |
| Fully adjusted model | 0.7797 | 0.00489 | 0.7702 | 0.7893 | 0.5595 | 0.5595 | 0.1951 |
| Underweight vs normal weight (Figure 2) | | | | | | | |
| Empty model | 0.5000 | 0 | 0.5000 | 0.5000 | 0 | . | 0 |
| Fully adjusted model | 0.7030 | 0.00546 | 0.6923 | 0.7137 | 0.4060 | 0.4061 | 0.1457 |

Table 2: ROC Contrast Test Results

| ROC Contrast Test Results | | | |
| --- | --- | --- | --- |
| Contrast | DF | Chi-Square | Pr > ChiSq |
| Overweight/obesity vs normal weight | | | |
| Reference = Empty model | 1 | 3272.4292 | <.0001 |
| Underweight vs normal weight | | | |
| Reference = Empty model | 1 | 1384.4075 | <.0001 |

Figure 1: ROC curve for Overweight/obesity vs normal weight

Figure 2: ROC curve for underweight vs normal weight

Figure 3: Diagnostics vs Predicted Probability for overweight/obesity vs normal weight comparison


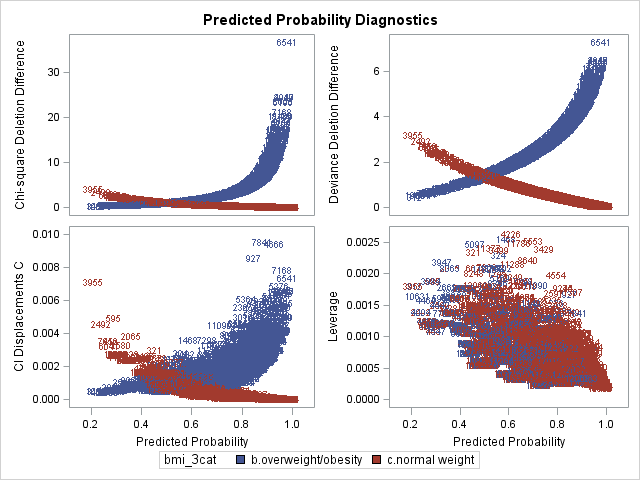

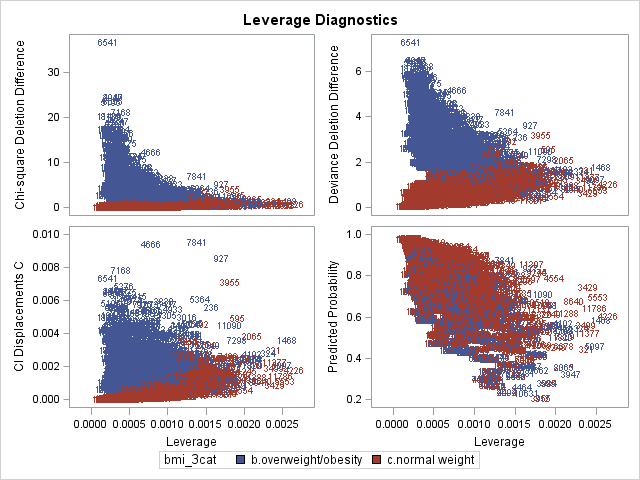


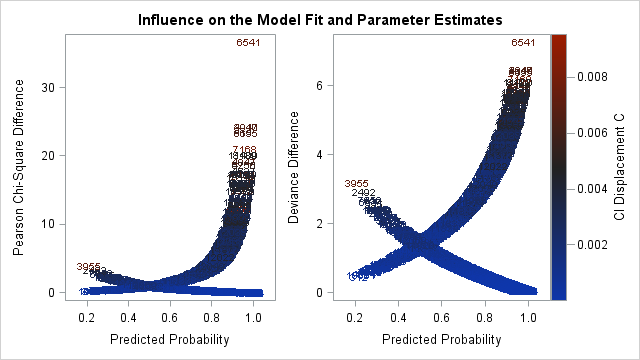


Figure 4: Diagnostics vs Predicted Probability for underweight vs normal weight comparison


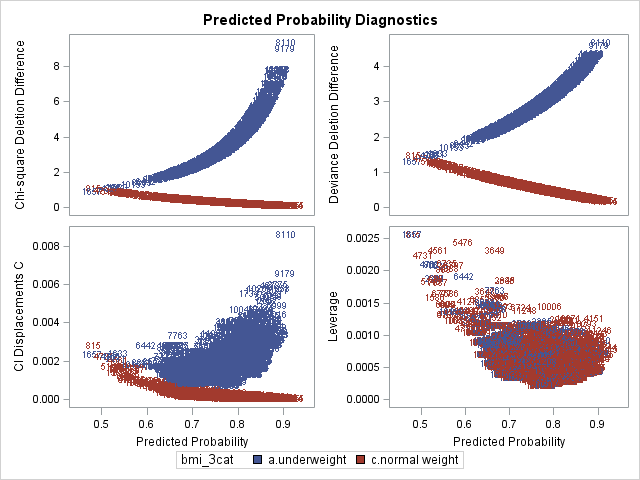

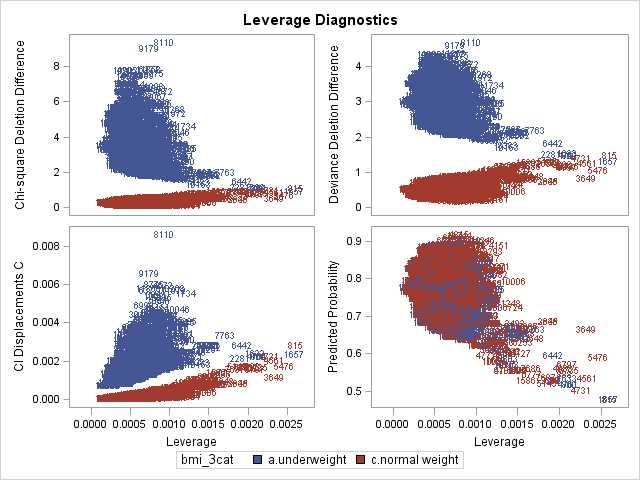


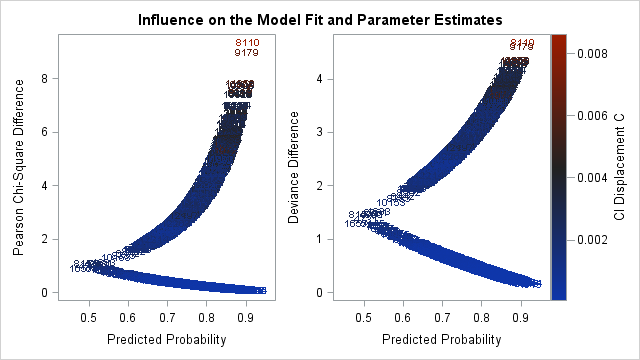


**Summary**

ROC curves for model fit, and diagnostic tests for influential observations and maximum likelihood shows overall good fit and few outlying observations.

**References**

1. Pregibon, D. (1981), “Logistic Regression Diagnostics,” **Annals of Statistics**, 9, 705–724.

### SAS. Example 72.6 Logistic Regression Diagnostics. Available from, <http://support.sas.com/documentation/cdl/en/statug/68162/HTML/default/viewer.htm#statug_logistic_examples06.htm> (Accessed 18 February 2018)
